# When Delay Becomes Danger: Sudden Progression of First-Degree to High-Grade Block

**DOI:** 10.1016/j.jaccas.2026.108037

**Published:** 2026-05-20

**Authors:** Perneet Powar, John Evans

**Affiliations:** Division of Cardiology, Department of Medicine, Highland Hospital, Oakland, California, USA

**Keywords:** atrioventricular conduction, complete heart block, diagnostic challenge, electrocardiography, first-degree heart block

## Abstract

**Background:**

While a first-degree block is benign, a high-grade block often reflects conduction failure requiring device placement.

**Case Summary:**

A 63-year-old man with a past medical history of hypertension, known first-degree atrioventricular block, and remote endocarditis presented with cellulitis and fatigue. Electrocardiogram(s) revealed alternating Mobitz 1 and high-degree blocks.

**Discussion:**

The patient was offered an electrophysiology study to evaluate the location of the block but elected for pacemaker placement. The sudden transition from a first-degree to 2:1 block is unusual for a vagal block, suggesting the possibility of a complete block with an isorhythmic junctional escape rhythm.

**Take-Home Messages:**

An abrupt transition between a first-degree and high-grade block should prompt evaluation for a 2:1 block with a shortened PR interval vs a complete heart block with an isorhythmic junctional escape rhythm. Exercise and other noninvasive measures can help determine chronotropic incompetence with lack of PR shortening and clue diagnosticians into an infranodal conduction disorder.

## Visual Summary

**Figure:**
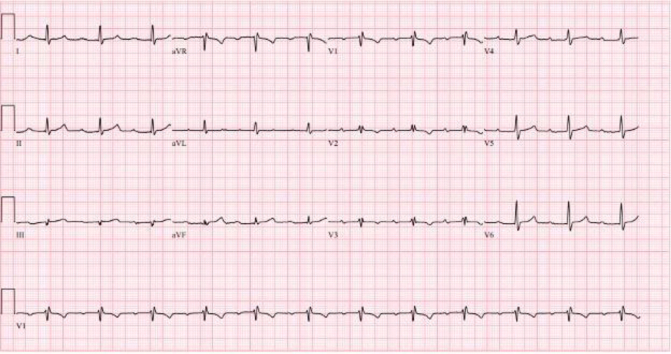

## Clinical vignette

**Figure 1:**
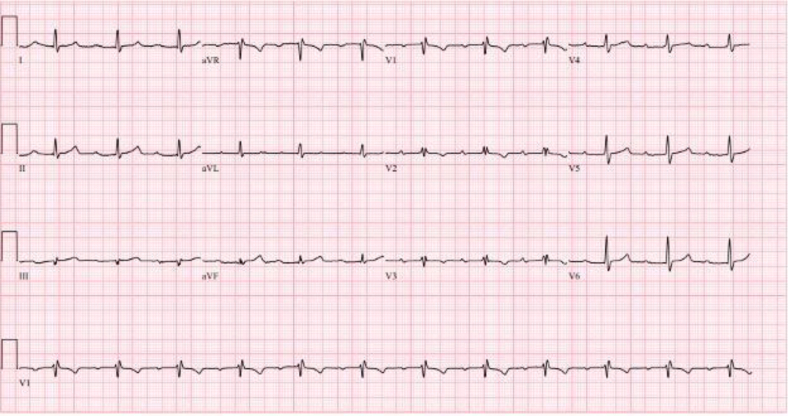
Baseline Electrocardiogram With a Prolonged PR Interval

**Figure 2:**
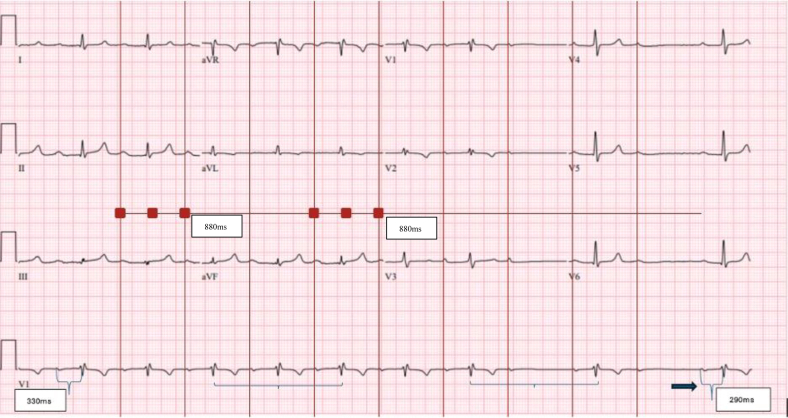
Repeat Electrocardiogram Demonstrating Fixed PP Intervals and Sudden Prolongation of VV and PR Intervals

A 63-year-old man with a past medical history of hypertension, cirrhosis of unknown etiology, known first-degree atrioventricular (AV) block (Figure 1), and remote endocarditis presented with lower extremity cellulitis and fatigue. Examination was notable for body mass index >50 and dropped beats on pulse check. Laboratory findings were notable for thrombocytopenia. Initial electrocardiogram (ECG) showed a Mobitz second-degree type 1 AV block. Repeat ECG was performed (Figure 2). An exercise study completed after this ECG demonstrated a fixed PR interval, with a maximum heart rate of 80-89 beats/min. An echocardiogram showed normal ejection fraction and mild biatrial dilation.

## Multiple-choice question

Which of the following is the most likely cause of the shortened PR interval of 290 milliseconds on the above ECG?

A. Decreased vagal tone
B. Junctional escape
C. Accessory pathway
D. Increased adrenergic tone

Choice A is incorrect. The conduction slows from 1:1 to 2:1 while the PP interval stays constant. Decreased vagal tone would be expected to continue 1:1 conduction with an increased heart rate.

Choice B is correct. This patient has a history of first-degree and Mobitz second-degree type 1 AV block but no prior shortened PR conduction as seen after the dropped beats on the second ECG. This is likely not a true PR interval and reflects a junctional escape in the setting of a high-grade AV block that is not associated with the preceding P-wave as supported by the fixed PP and the changing VV intervals.

Choice C is incorrect. There is no evidence of pre-excitation on this patient's ECG to suggest the presence of an accessory pathway.

Choice D is incorrect. Increased adrenergic tone would still reflect as 1:1 conduction instead of 2:1, and the sinus rate would also be expected to increase.

This patient's ECG initially shows 1:1 conduction with a long PR interval that suddenly appears to progress to a high-grade block. Differential includes sudden 2:1 block (type 1 or type 2) with a shorter PR interval and a complete heart block with an isorhythmic junctional escape rhythm. The presence of a long PR interval with 1:1 conduction and long cycle Wenckebach are typically indicative of an AV node conduction problem; therefore, it is uncommon to have a sudden transition to a 2:1 block.^1^ Based on this ECG alone (second-degree block with narrow QRS complexes), the conduction issue should be at the level of the AV node. However, the presence of RSR′ in V_1_ is concerning for an infra-Hisian block despite the narrow QRS complexes. In addition, with exercise there was no shortening of the PR interval, further suggesting an infranodal conduction problem; heart rate only increased to 80-89 beats/min, revealing chronotropic incompetence and likely underlying sinoatrial node dysfunction as well. We cannot exclude progression to a third-degree AV block with an isorhythmic junctional escape rhythm. Alternatively, this could be infra-Hisian Wenckebach, with the long RR interval allowing for faster conduction in the His-Purkinje system. The patient was offered an electrophysiology study to further evaluate the origin of the conduction problem vs proceeding straight to pacemaker placement due to his symptoms and chronotropic incompetence. The patient elected to proceed with pacemaker placement.

## Funding Support and Author Disclosures

The authors have reported that they have no relationships relevant to the contents of this paper to disclose.

**Take-Home Messages**

• An abrupt transition between a first-degree and high-grade block should prompt evaluation for a 2:1 block with a shortened PR interval vs a complete heart block with an isorhythmic junctional escape rhythm.
• Exercise and other noninvasive measures can help determine chronotropic incompetence with the lack of PR shortening and clue diagnosticians into an infranodal conduction disorder.
